# Preliminary Development of a Health Education Program to Improve Psychological Distress Among Patients with Esophageal Cancer and Their Partners: A Narrative Review

**DOI:** 10.3390/healthcare13172210

**Published:** 2025-09-03

**Authors:** Meng Wei, Caryn Mei Hsien Chan, Azlina Yusuf, Maziah Ahmad Marzuki

**Affiliations:** 1 Department of Nursing, Faculty of Medicine, Universiti Kebangsaan Malaysia, Cheras, Kuala Lumpur 56000, Malaysia; p117780@siswa.ukm.edu.my; 2 School of Nursing, Hebei Medical University, Shijiazhuang 050017, China; 3 Center for Community Health Studies (ReaCH), Faculty of Health Sciences, Universiti Kebangsaan Malaysia, Jalan Raja Muda Abdul Aziz, Kuala Lumpur 50300, Malaysia; caryn@ukm.edu.my; 4 School of Health Sciences, Universiti Sains Malaysia, Kubang Kerian 16150, Kelantan, Malaysia; azlinayusuf@usm.my

**Keywords:** behavioral activation, diet, esophageal cancer, physical activity, psychological distress

## Abstract

**Background**: The diagnosis and treatment of esophageal cancer often leads to complex and long-lasting psychological distress, such as anxiety and depression, in patients and their partners. This psychological distress can not only potentially worsen the poor prognosis of the disease, but also reduce health-related quality of life by affecting the patient’s ability to function and enjoy life. **Objectives**: These preliminary data were collected to identify the components required for the development of a health education program pertaining to improving psychological distress. **Methods**: A narrative review. **Results**: Two components, diet and physical activity, were identified as important factors for the well-being of esophageal cancer patients and their partners with psychological distress. Moreover, behavioral activation was assumed to be an effective approach for assisting esophageal cancer patients’ behavioral compliance with the given dietary intake and physical activity practices program. **Conclusions**: A health education program based on the above components (diet and physical activity) with a behavioral activation approach could be developed as a guideline to address the problem of psychological distress among esophageal cancer patients and their partners. However, these conclusions should be treated with caution, given that the findings have not yet been empirically tested. Further rigorous studies are required to confirm their effectiveness and determine which program components may be most effective in improving outcomes.

## 1. Introduction

Esophageal cancer is a prevalent form of upper gastrointestinal cancer that significantly contributes to the global health burden, particularly in developing countries. According to the GLOBOCAN 2020 report published by the International Agency for Research on Cancer, the incidence of esophageal cancer has declined to the seventh most common cancer and the sixth most deadly cancer globally in recent decades; however, mortality continues to increase [1,2]. It is estimated that deaths from esophageal cancer are expected to increase by approximately 68% by 2040 [3]. Owing to this, along with its high rates of morbidity, mortality, and complications, psychological distress is at high risk of being developed or exacerbated, even when compared to other forms of cancer patients [4].

According to the National Comprehensive Cancer Network, cancer-related psychological distress is defined as ‘an unpleasant emotional experience’ that is psychological, social, and/or spiritual in nature. This may affect patients’ ability to cope with cancer and its physical symptoms and treatment [5]. Distress exists on a continuum, ranging from normal feelings of vulnerability, sadness, and fear to more severe problems such as depression, anxiety, panic, social isolation, and existential and spiritual crises [6]. As patient-centered care has developed, these crises have emerged as the psychological distress symptoms most frequently reported by patients with esophageal cancer and their partners [4].

The relationship between psychological distress and esophageal cancer has been a topic of great interest. Liu Y, Pettersson E, Schandl A, Markar S, Johar A, and Lagergren P [7] reported that 11.5% of patients with esophageal cancer experienced clinically significant psychological distress, with this proportion increasing to 18.8% at 1.5 years and to 25.0% at 2 years post-surgery. The high prevalence and longitudinal increase in patient-reported psychological distress indicate the unmet demands of timely and effective psychological health education after esophageal cancer treatment [8]. Previous studies have shown that anxiety and depression may worsen cancer-related symptoms, reduce health-related quality of life, including the ability to perform daily routines on their own, and even cause poor survival rates for esophageal cancer patients [9,10]. Previous studies have identified the beneficial effects of health education programs on improving the psychological distress of cancer patients. However, most of these studies focused on prostate or breast cancer patients. Fewer studies have been conducted on programs for patients with esophageal cancer, and the evidence on their effectiveness is insufficient. Therefore, there is a need for the development of a health education program that assists patients with esophageal cancer and their partners in managing psychological distress, thereby promoting their well-being and functionality, as well as enjoying life.

## 2. Methods

A narrative review is a qualitative synthesis of literature that summarizes and interprets the existing evidence using a flexible, non-systematic approach [11]. It provides a comprehensive overview of the current knowledge, identifies patterns and trends, and establishes a context for future research. The synthesis of results may incorporate a wider range of study types and perspectives and draw on diverse evidence to highlight key debates, gaps, and future directions.

### 2.1. Research Question

This review, using a narrative summary, determined what components are needed to develop a health education program aimed at improving psychological distress in patients with esophageal cancer and their partners.

### 2.2. Search Strategy

Six electronic databases (MEDLINE, CINAHL, Web of Science, Embase, PsycINFO, and the Cochrane Library) were searched from their inception until 30 April 2025. The search strategy combined medical subject headings and free-text terms, using Boolean operators (AND and OR). Keywords such as “esophageal cancer, psychological distress, program, intervention, diet, physical activity” were used. The search strategy is presented in Tables S1–S6 (see Supplementary Materials).

### 2.3. Inclusion and Exclusion Criteria

Only peer-reviewed full-text journal articles written in English were considered, with no geographic location restrictions. Furthermore, the reference lists of the selected articles were reviewed to identify additional eligible studies. We included original research articles, clinical trials, observational studies, systematic reviews, and expert consensus guidelines on interventions for psychological distress in patients with esophageal cancer. Studies reporting on more than one type of cancer or focusing solely on clinical treatment were excluded. In addition, protocols, trial registries, and conference abstracts or proceedings were excluded.

### 2.4. Study Selection and Data Extraction

To minimize selection bias, two authors screened the titles and abstracts independently after deduplication. The full texts were then evaluated according to the inclusion criteria. After reading the full texts, the eligible studies were finally selected. Rather than seeking to exhaustively cover all existing studies, a narrative review aims to screen them based on topic relevance, methodological rigor, and contribution to the research question. This was judged independently by two reviewers, who also extracted the data. Any discrepancies were resolved through discussion and negotiation, and a third reviewer was consulted if necessary.

### 2.5. Quality Assessment

As this is a narrative review, no formal risk of bias assessment tool was applied. Only peer-reviewed studies were considered to ensure the inclusion of high-quality, evidence-based sources. Including studies from diverse countries and regions was intended to ensure representativeness and minimize publication bias.

### 2.6. Data Synthesis

The final selection of articles was based on their relevance to the topic and the quality of the evidence. Data extraction was divided into four thematic areas to reflect key challenges, gaps, and future directions in improving health education on psychological distress among esophageal cancer patients and their partners.

## 3. Results

### 3.1. Psychological Distress of Esophageal Cancer Patients

Timlin D, McCormack JM, Kerr M, Keaver L, and Simpson EE [12] defined “diet behavior” as a multifaceted dimension, including but not limited to “diet intake”, “eating behavior”, “eating habits”, and “food choice”. Restricted dietary behavior and the resulting long-term weight loss are known causes of psychological distress among patients with esophageal cancer and their partners. Owing to the complicated therapeutic process of treating esophageal cancer, it is often difficult for patients to return to normal diet patterns [13]. Dysphagia is one of the most prevalent symptoms prior to diagnosis, more than a third of patients reported experiences of it persisting for up to five years following surgery, and can cause a great deal of psychological distress [14]. Many patients have also described experiencing food intolerances after surgery, resulting in changes to their diet, weight loss, and a recovery process similar to their pre-treatment symptoms [4]. Re-experiencing these pretreatment symptoms compounds the psychological distress associated with the disease. In addition, enteral/tube feeding, delivering nutrition directly into the gastrointestinal tract is a standard procedure after esophagectomy, has also been identified as an independent risk factor for depression [4]. Patients often have to relearn the biofeedback mechanisms of satiety, nausea, and vomiting for regular use and requires professional care as well as a formula-based diet. Consequently, patients may experience social isolation, which can negatively impact their mood and reduce their support mechanisms. This potentially leads to depression and suicidal thoughts.

Guidelines have consistently suggested a variety of healthy diet behaviors for cancer survivors, including improving the intake of fruits, vegetables, whole grains, and soybeans while limiting the intake of red meat, processed meat, alcohol, sugar, salt, and dietary supplements [13]. Although the guidelines have provided consistent recommendations on healthy diet behaviors, numerous studies have shown that people often have unfavorable adherence to the recommended diet behaviors [15]. For example, as suggested by a meta-analysis focusing on the Western population (*n* = 51 studies with 2,620,586 participants), cancer patients generally have good adherence to the recommendations on alcohol (83%) and sugar (69%), but poor adherence to the recommendations on whole grains (31%), fruits and vegetables (34%), salt (36%), and processed meats (47%) [16].

Another point that must be emphasized is the connection between physical activity and psychological distress. Physical activity is defined by the U.S. Department of Health and Human Services (HHS) as any movement in which skeletal muscles are used in terms of type, frequency, duration and intensity, as well as the physical environment in which it occurs and more energy than at rest is used up [13]. Pain and limited physical activity after esophagectomy are known risk factors for psychological distress [8,17]. For patients with esophageal cancer and their partners, worse tumor conditions mean larger parts of resection during surgery and worse physical dysfunction after surgery [4]. Increasing physical activity has been demonstrated to be an effective and safe strategy to improve psychological distress, specifically anxiety and depression, in populations diagnosed with different cancers [18,19]. Physical activity can effectively promote the recovery of somatic functions and reduce sedentary behavior, thereby relieving anxiety and depression [20]. Moreover, compared with physically inactive cancer patients, cancer patients who engage in physical activity for more than ≥150 min per week are 61% less likely to report serious psychological distress [21]. This matter is supported by an international roundtable discussion which also concluded that exercise could reduce sedentary behavior and reduce the risk of developing psychological distress in cancer patients [22].

Previous studies have shown that the physical activity recommended by the American Cancer Society can effectively promote the recovery of somatic functions and relieve anxiety and depression [23]. However, adherence to physical activity is also a challenge for patients with esophageal cancer and their partners. Owing to limited disease knowledge, insufficient social support, lack of time, and poor psychological status, the level of physical activity among esophageal cancer patients and their partners is low [24]. Moreover, previous studies have indicated that a large portion of patients cannot maintain the recommended physical activity level and return to sedentary behaviors over time after being discharged [25].

There is evidence that diet and physical activity are closely linked to psychological distress and health-related quality of life in esophageal cancer patients during treatment [26,27]. The results suggested that patients with esophageal cancer and their partners could be receptive to explicit attention to diet and physical activity as part of psychosocial care. Despite reported barriers to compliance with healthy diets and physical activity in patients with esophageal cancer and their partners, interest in obtaining more information and participating in them is still high. At the same time, previous research on health education programs related to compliance behaviors to healthy diets and physical activity among patients with esophageal cancer and their partners to improve psychological distress remains very limited.

### 3.2. Behavioral Activation Approach for Health Education Programs

Behavioral activation is a brief, structured psychosocial intervention that aims to alleviate depression by gradually increasing participation in enjoyable, meaningful activities and reducing avoidance and rumination [28]. As a third-generation behavioral therapy, it is rooted in the biological basis of behavior and emphasizes the link between behavior and mood [29]. Peter Lewinsohn first articulated the approach in the 1970s, proposing that clients should be assisted in engaging in behaviors that they find pleasurable or fulfilling—in other words, positive reinforcement [30,31].

What should we know about behavioral activation? First, the key aspects of behavioral activation are as follows: (a) activity monitoring. This refers to tracking behaviors and their impact on mood to identify patterns. (b) Activity scheduling. This refers to planning and engaging in activities that are likely to improve mood. (c) Identifying and addressing barriers. This involves recognizing and overcoming obstacles that prevent engagement in desired activities. (d) Focusing on positive reinforcement. This involves increasing activities that expose the individual to positive environmental contingencies; (e) Reducing avoidance behaviors. This involves reducing behaviors that limit access to positive reinforcement, such as inactivity or staying indoors, and increasing activities that promote movement [32].

Moreover, the mechanism of behavioral activation functions by helping individuals understand how their behaviors influence their mood and encouraging them to engage in activities that can improve their overall well-being. It is a structured, time-limited approach that can be used as a standalone treatment or alongside other therapies to improve an individual’s emotional suffering [33]. Behavioral activation holds that depression often arises from a lack of positive reinforcement in one’s environment. Hua C, Yao X, and Piatt JA [34] reported that individuals with depression tend to withdraw from activities they once enjoyed, leading to reduced positive experiences, which in turn exacerbate depressive symptoms, creating a vicious cycle. Behavioral activation aims to break this cycle by encouraging individuals to engage in meaningful and rewarding activities, thereby improving mood and overall well-being [35].

In addition, the facts clearly show the benefits that can be obtained through behavioral activation methods, namely, (a) improved mood, by engaging in enjoyable and meaningful activities, individuals can experience an increase in positive emotions; (b) increased motivation and engagement, since behavioral activation can help individuals regain a sense of purpose and find enjoyment in their lives; (c) reduced avoidance behaviors, by addressing barriers to activity, individuals can reduce avoidance behaviors that contribute to low mood; and (d) improved overall well-being, since behavioral activation can help individuals build healthier habits and improve their quality of life [36]. Watson R, Harvey K, Pass L, McCabe C, and Reynolds S [37] reported that behavioral activation significantly reduced depressive symptoms and improved mood and that improvements in positive emotions were associated with increased motivation and engagement in positive behaviors. Ure SL, Gill C, Evans T, Windsor TD, Scott JE, Walker R, Luszcz MA, and Mazzucchelli TG [38] showed that behavioral activation can reduce avoidance behaviors and promote healthier habits, thereby improving overall well-being. In addition, behavioral activation can reduce time costs, thus ensuring smooth implementation of the program [39].

Behavioral activation can be adapted for esophageal cancer patients through gradual activity scheduling. This involves starting with small, achievable goals, such as 5 min walks, and incrementally increasing the duration and intensity as the patient’s physical condition improves. This approach can help to address treatment-related fatigue, pain, and other side effects. Pleasant activities are adapted to physical limitations; for example, gentle stretching or low-exertion social interactions (such as video calls) are recommended instead of strenuous exercise. Barriers such as nausea, dysphagia, or anxiety can be overcome using pacing strategies (breaking tasks down into smaller steps), collaborating with healthcare teams on pain management, relaxation techniques such as deep breathing, nutritional support, cognitive restructuring to challenge negative beliefs, and encouraging social support. Behavioral activation should be tailored to the individual needs and preferences of each patient, considering their specific diagnosis, treatment plan, and personal circumstances. Close collaboration with the patient’s healthcare team is often necessary, including oncologists, nurses, dieticians, caregivers, and partners. These approaches can help people with esophageal cancer to maintain or improve their quality of life, manage their symptoms, and reduce their risk of depression and other psychological distress.

Therefore, researchers have aimed to integrate behavioral activation as a new approach into health education programs, focusing on diet and physical activity for esophageal cancer patients and their partners, which can be used to create a holistic approach to improve diet and physical activity by encouraging behavioral compliance and improving psychological well-being [40]. By incorporating behavioral activation principles, patients and partners can be encouraged to engage in structured, goal-oriented activities such as setting specific dietary goals, scheduling regular exercise sessions, and participating in shared physical activities. These actions aim to counteract avoidance behaviors and reinforce positive behaviors, which can reduce psychological distress. The expected outcomes include reduced psychological distress, improved adherence to health recommendations, and enhanced quality of life for both patients and their partners [41]. This finding demonstrates the value of combining behavioral activation with health education in cancer care. In addition, previous studies on the use of behavioral activation have been conducted in studies related to emotional disorders [39] and have not yet been found to be useful in health programs for esophageal cancer patients. This finding indicates that this approach is highly important for this health program development study.

### 3.3. Health Education Programs as Psychological Distress Improvement Methods for Esophageal Cancer Patients

In the critically reviewed literature, there are several studies of health education programs on diet and physical activity for esophageal cancer patients and their partners. The following content is presented: (1) What are the current diet and physical activity programs for patients with esophageal cancer and their partners? (2) What are the characteristics of these programs? (3) What are the effects of different programs on psychological distress?

#### 3.3.1. A Rehabilitation Program: 12-Week Brisk Walking and Diet Education Program

Chen, Lin YY, Wu YC, Huang CS, Hsu PK, Chien LI, Lin YJ, and Huang HL [20] conducted a pilot randomized controlled trial to examine the impacts of a rehabilitation program involving brisk walking and diet education on anxiety and depression among patients with esophageal cancer. According to self-efficacy theory, professionals can enhance patients’ sense of self-efficacy by sharing their knowledge and skills with them, as well as offering positive reinforcement throughout their recovery. This program combined moderate-intensity brisk walking (40 min, 3 times/week) with comprehensive diet education, which was delivered via a booklet and weekly phone consultations. Forty-four participants were randomized to either the intervention group (program + weekly telephone consultation) or the control group (usual care, including providing nutrition information and follow-up) at a 1:1 ratio, with 22 participants in each group. Patients were referred to the researchers by physicians from the outpatient department. The Hospital Anxiety and Depression Scale (HADS) was used to assess anxiety and depression at baseline and three months post-intervention. Pretest data were collected from patients during their outpatient visits to the chest surgery department. Post-test data were collected via mail. The results indicated a mean exercise completion rate of 73.74% in the intervention group, with 16 participants completing a minimum of 70% of sessions, and no adverse effects were detected in the exercise program. Compared with the control group, the intervention group exhibited a marginal but statistically significant improvement in anxiety scores. This pilot study suggests that the combined exercise and diet education program is a feasible and low-cost intervention with the potential to alleviate anxiety in esophageal cancer patients. This study’s success highlights the potential of nonpharmacological approaches for improving the psychological well-being of this patient population.

#### 3.3.2. Mobile Health-Supported Comprehensive Intervention Model (CIMmH) Program: 12-Week Nutritional, Exercise, and Psychosocial Support

Cheng C, Ho RTH, Guo Y, Zhu M, Yang W, Li Y, Liu Z, Zhuo S, Liang Q, Chen Z, Zeng Y, Yang J, Zhang Z, Zhang X, Monroe-Wise A, and Yeung S-C [42] designed the first comprehensive intervention model supported by a mobile health system (CIMmH) delivered on the WeChat platform, which provides nutrition, exercise, and psychological support to patients post-esophagectomy with aimed to assess the feasibility and safety of 12-week of the program. This prospective pilot study is an innovative program, delivered through the WeChat platform and in-person hospital visits, integrates three key components: nutrition guidance, physical exercise, and psychological psychosocial support. Nutrition support involves individualized plans tailored to three distinct postsurgical phases, provided through face-to-face consultations with a nutritionist and supplemented with online educational materials and videos on the WeChat platform. The exercise protocol included inspiratory muscle training, walking exercises, and Baduanjin qigong, promoting both physical recovery and respiratory function. Psychosocial support was adapted from mindfulness-based cancer recovery (MBCR) principles and delivered through the WeChat platform with ongoing monitoring and support. Twenty patients participated in this prospective, single-arm pilot study, with assessments at baseline and at 1 month and 3 months post-surgery. Sixteen patients (80%) completed the program. Depressive and anxiety symptoms, measured via the Patient Health Questionnaire-9 (PHQ-9) and the Generalized Anxiety Disorder Questionnaire-7 (GAD-7), improved from 1 to 3 months post-surgery, exceeding the recovery rate of traditional programs. The study concluded that the CIMmH model proved feasible and well received, offering a promising approach to postsurgical care, which is particularly beneficial for patients with limited access to frequent hospital visits or who reside in remote areas. The findings support further research into broader implementation of this mHealth-based model, namely, the CIMmH program.

#### 3.3.3. Self-Management Program (OptiMal): 6-Week Exercise and Physical Activity & Healthy Eating

King E, Algeo N, and Connolly D [43] assessed the feasibility of OptiMal, a six-week, group-based self-management program for esophageal cancer survivors aimed at improving mood. On the basis of self-management support and self-efficacy theories, OptiMal’s six 2.5 h sessions covered exercise, physical activity, and healthy eating strategies to manage persistent symptoms such as fatigue and distress. Each session included educational components, facilitated peer discussion, and goal-setting activities to promote self-directed management. A mixed-methods design, including pre- and postintervention Hospital Anxiety and Depression Scale (HADS) assessments and qualitative feedback, was used to evaluate the program’s feasibility in Ireland. Fourteen of the twenty-four eligible participants completed OptiMal (89.3% attendance), demonstrating high levels of engagement and adherence. While immediate post program anxiety or depression scores showed no significant improvement, statistically significant reductions in anxiety and depression were observed three months later, suggesting a delayed but substantial positive impact. The participants found the program’s content, format, and delivery relevant and appropriate. These findings suggest the feasibility and potential effectiveness of OptiMal for esophageal cancer survivors. Future research should use robust, controlled study designs with larger sample sizes to investigate the efficacy of OptiMal for esophageal cancer survivors.

#### 3.3.4. Physical Exercise Following the Esophageal Cancer Treatment (PERFECT) Program: 12-Week Aerobic and Resistance Exercise

Van Vulpen JK, Hiensch AE, van Hillegersberg R, Ruurda JP, Backx FJG, Nieuwenhuijzen GAP, Kouwenhoven EA, Groenendijk RPR, van der Peet DL, Hazebroek EJ, Rosman C, Wijnhoven BPL, van Berge Henegouwen MI, van Laarhoven HWM, Siersema PD, and May AM [44] conducted the PERFECT multicenter randomized controlled trial (RCT) to explore the effects of a 12-week program combining aerobic and resistance exercises for anxiety and depression on patients with esophageal cancer who have undergone surgery with curative intent. Given that the design of this program is closely integrated with daily life, this study can not only provide evidence for the effects of it on respondents, but also serve as a reference for potential implementation strategies. The exercise program consisted of two weekly, supervised sessions combining aerobic and resistance training, which were individualized to each patient’s fitness level. The sessions lasted one hour and included warm-up, exercise, and cool-down periods. The participants in the exercise group were also encouraged to engage in at least 30 min of daily moderate-intensity aerobic activity on non-intervention days as referring to guidelines from the World Cancer Research Fund/American Institute for Cancer Research (WCRF/AICR). Outcomes were assessed via the Hospital Anxiety and Depression Scale (HADS). One hundred and twenty patients were randomly allocated to one of two groups, either an exercise intervention group or a control group who received usual care. Among them, 61 were in the intervention group, and 59 were in the control group. The results revealed that ninety-eight participants completed the 12-week program, indicating high adherence rates. Significant improvements in cardiorespiratory fitness were observed in the exercise group at the 12-week mark, although these gains diminished by the 24-week follow-up. Importantly, no significant between-group differences were found in anxiety or depression scores. It was concluded from the study that the supervised exercise program combining aerobic and resistance training was both safe and feasible for esophageal cancer patients post-esophagectomy. While it provides short-term benefits for cardiorespiratory fitness, further research is needed to explore its long-term effects on mental well-being.

#### 3.3.5. Integrated Medical and Nursing Intervention Program: Dietary Guidance and Psychological Nursing During Hospitalization

Wang ZY, Cheng YQ, Li JJ, and Hu XY [45] conducted a prospective study to investigate the effects of an intervention of integrated medical–nursing model for improving the psychological mood of patients with esophageal cancer who undergoing radiotherapy. They selected 78 patients with esophageal cancer undergoing radiotherapy in the hospital and randomly divided them into two groups: a control group and an intervention group. In the control group (*n* = 39), doctors were responsible for cancer therapy, and nurses provided usual inpatient care. In the intervention group (*n* = 39), patients received an integrated medical–nursing model that physicians and nurses joined forces to provide dietary guidance to patients, and if necessary, a full-time nutritionist was invited to develop a dietary plan for patients. In addition, medical staff often provide psychological care to patients, inviting professional psychological counsellors to provide targeted psychological counselling. A WeChat group was also set up to facilitate communication. Instruments of the Hamilton Anxiety Rating Scale (HAMA) and the Hamilton Depression Rating Scale (HAMD) were used to compare the psychological status of the two groups of patients before the intervention and at discharge. The results revealed that all participants in the intervention and control groups completed the programs, with a completion rate of 100%. Compared with the preintervention scores, the anxiety and depression scores were lower in both groups at discharge and lower in the intervention group than in the control group. This study suggests that an integrated medical and nursing intervention can reduce unhealthy emotions and improve nutritional status and is worthy of clinical application.

### 3.4. Application of Components from Previous Research in Health Education Programs

The above studies are health education programs that use various program components with different outcomes that impact or do not impact psychological distress issues among patients with esophageal cancer and their partners. Table 1 presents a summary of previous studies pertaining to health education programs and psychological distress.

## 4. Discussion

Referring to Table 1, the results of the document analysis of these studies provide ideas for current researchers to develop a new program that consists of diet and physical activity components to improve psychological distress, but it may remain noncompliant with behavioral practices. Moreover, behavioral activation, as a new approach, will be introduced in the program to help researchers increase behavioral adherence to planned healthy eating and physical activity plans among study respondents. Researchers believe that this behavioral activation will improve and have a more positive effect on dealing with psychological distress issues. This is because the results of previous studies on mood/emotional disorders patients showed that behavioral activation can help individuals regain a sense of purpose and find enjoyment in their lives to increase motivation and engagement in activities that can improve their overall well-being [28]. Therefore, researchers hope that similar results will be obtained when this behavioral activation approach is used to develop a health education program for esophageal cancer patients and their partners who are experiencing psychological distress. On the other hand, researchers assume that the expected outcome of the study is that reducing depression will improve psychological distress, motivate behavioral compliance and increase an individual’s health-related quality of life, including functionality and enjoyment of life, by using the behavioral activation approach in the proposed health education program.

To strengthen the theoretical basis of our model, we now situate it within Social Cognitive Theory. This theory emphasizes the interplay between individual factors, behavior, and the environment in shaping health behaviors. There is evidence to support both directions of the relationship. For example, improved well-being can motivate individuals to adopt healthier lifestyles. Conversely, adopting positive lifestyle changes (such as improving diet and increasing exercise) can lead to an enhanced mood, reduced stress, and an improvement in overall well-being. Additionally, the Dual Continua Model posits that psychological distress and well-being are distinct, yet interconnected, facets of mental health. Distress is often associated with negative emotions, maladaptive behaviors, and difficulty coping with challenges. Well-being, on the other hand, encompasses positive emotions, life satisfaction, and a sense of purpose. While distinct, the two concepts are also intertwined. It is possible to experience high levels of distress alongside positive well-being, or vice versa. However, these pathways remain hypothetical and await rigorous empirical validation.

**Figure 1:**
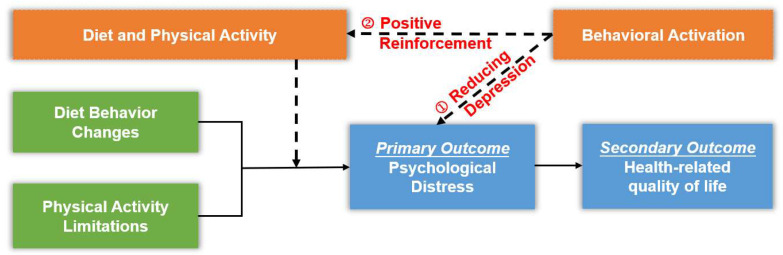
Conceptual framework of program development.

Overall, as shown in Figure 1, the dependent variable for this study is psychological distress, and the independent variables are diet and physical activity with behavioral activation approaches. Previous research has identified limited physical activity and dietary behavior change as core risk factors for psychological distress in patients with esophageal cancer. These factors may also increase the caregiving burden for partners, leading to psychological distress. Therefore, it is hypothesized that physical activity and diet are the primary predictors of psychological distress. Improving diet and physical activity can consequently improve psychological distress for couples with esophageal cancer. Additionally, behavioral activation may improve psychological distress partially by reducing depression and partially by increasing patients’ motivation (positive reinforcement) to follow healthy eating and physical activity.

### 4.1. Limitations

There are several limitations to this review that should be noted.

Firstly, searching for published literature was restricted to English, which may lead to publication bias. People from different cultural backgrounds respond differently to negative experiences. Therefore, it is crucial to develop health education programs based on specific cultural contexts. Further research is necessary to explore the effectiveness of culturally specific health education programs for patients with esophageal cancer.

Secondly, the studies included were heterogeneous, making it difficult to compare the effectiveness of the interventions. The intervention characteristics of the different studies varied in terms of components, delivery, and duration, and the participants were at different stages of treatment. Therefore, the results should be interpreted cautiously for further recommended that future researchers who implement randomized controlled designs rigorously are able to report more specifics in line with reporting guidelines, thereby ensuring the quality of reporting.

Thirdly, as this review is based entirely on the existing literature (secondary data), the results are speculative and must be tested by future empirical research. While the aim of this review is to synthesize existing research and offer new insights rather than verify hypotheses, the limitations resulting from this approach should still be acknowledged (e.g., the reliability of conclusions depends on the quality of the existing literature). Only a small number of studies with small numbers of participants were included, and these were inadequately powered to detect intervention effects, which may have resulted in weaker evidence. In addition, several studies exhibited methodological limitations and were deemed to be at a high risk of bias.

Lastly, although the limited existing research provides preliminary evidence of the potential of behavioral activation in oncology, many methodological and practical challenges remain regarding its implementation in this context. More targeted research in this area is necessary in the future.

### 4.2. Implications for Clinical Practice and Research

This review highlights the psychological distress experienced by patients with esophageal cancer and their partners. It encourages healthcare professionals to use behavioral activation to improve patient outcomes, re-engage individuals in life, and deliver more efficient care. However, only a small number of studies were identified in this review, so more research should be conducted to investigate the effects of behavioral activation on esophageal cancer patients and their partners within specific cultural and geographical contexts. Additionally, future studies could examine the changes in psychological symptoms (e.g., stress, anxiety, and psychological distress) following behavioral activation interventions in more depth. Furthermore, none of the studies addressed the cost-effectiveness, adaptability, or optimal clinical implementation of behavioral activation for the growing population of cancer patients. Behavioral activation may encourage healthy behaviors in esophageal cancer patients and their partners, thereby improving their well-being. Understanding how it improves depression and reduces distress could inform targeted interventions.

## 5. Conclusions

In conclusion, the psychological distress experienced by esophageal cancer patients is closely linked to diet challenges and physical activity limitations. Drawing on components from prior research, health education programs focused on diet and physical activity were intended to address psychological distress issues. Integrating behavioral activation approaches into a health education program further provides a structured framework to address these challenges. Therefore, the development of a health education program requires components related to diet and physical activity in combination with behavioral activation approaches. The proposed health education program is expected to improve behavioral compliance among patients with esophageal cancer and their partners and to enhance their psychological distress and health-related quality of life. It is recommended that a structured health education program be developed and integrated into daily home care to further prevent psychological distress issues for patients with esophageal cancer and their partners. However, caution should be exercised when applying these findings, given the limited number of studies included and the risk of bias. The proposed health education program is only a preliminary construction and has not yet been empirically tested. Its effectiveness remains to be verified. More rigorous research could be conducted in the future to confirm not only the effectiveness of the proposed health education program, but also, more importantly, to examine which components might be most effective in improving physical and psychological well-being among esophageal cancer patients.

## Figures and Tables

**Table 1:** Summary of previous studies.

NO.	Previous Studies	Program Components			Delivery	Duration
		Diet	Physical Activity	Psychology		
1	Chen et al. (2022) [20] Rehabilitation program: brisk walking and diet education program	√	√	-	Face-to face and telephone consultations	12 weeks, three times a week
2	Cheng et al. (2020) [42] A mHealth-supported Comprehensive Program Model (CIMmH) program: nutritional, exercise, and mindfulness as psychological support	√	√	√	Face-to face and WeChat platform	12 weeks, divided into five stages
3	King et al. (2023) [43] A self-management program (OptiMaL): exercise and physical activity and healthy eating	√	√	-	Face-to face	6 weeks, Once a week
4	Van Vulpen et al. (2021) [44] The Physical ExeRcise Following Esophageal Cancer Treatment (PERFECT) program: Aerobic and resistance exercise	-	√	-	Face-to face	12 weeks, twice a week
5	Wang et al. (2021) [45] Integrated medical and nursing intervention program: dietary guidance and psychological nursing	√	-	√	Face-to face and WeChat platform	During hospitalization

## Data Availability

The original contributions presented in this study are included in the article. Further inquiries can be directed to the corresponding author.

## Supplementary Materials

The following supporting information can be downloaded at https://www.mdpi.com/article/10.3390/healthcare13172210/s1, Tables S1–S6: Search strategy of key databases.
