# Supplementary material for: Preliminary Development of a Health Education Program to Improve Psychological Distress Among Patients with Esophageal Cancer and Their Partners: A Narrative Review
Source: Healthcare (Basel). 2025 Sep 3;13(17):2210. doi: 10.3390/healthcare13172210 (PMC12428563; doi:10.3390/healthcare13172210)
Supplement: Supplementary file 1 [file healthcare-13-02210-s001.zip › healthcare-3732144-supplementary.pdf]

Table S1 Search strategy of MEDLINE (via PubMed)		
**Number**	**Query**	**Results**
#1	(esophageal neoplasms[MeSH Terms]) OR (esophagus[MeSH Terms])	106,789
#2	(esophageal cancer[Title/Abstract]) OR (oesophageal cancer[Title/Abstract])	30,424
#3	#1 OR #2	114,919
#4	(diet[MeSH Terms]) OR (exercise[MeSH Terms])	574,213
#5	((((((((((diet*[Title/Abstract]) OR (nutrition*[Title/Abstract])) OR (eating[Title/Abstract])) OR (food[Title/Abstract])) OR (physical activit*[Title/Abstract])) OR (exercise*[Title/Abstract])) OR (walk*[Title/Abstract])) OR (training[Title/Abstract])) OR (rehabilitation[Title/Abstract])) OR (lifestyl*[Title/Abstract])) OR (behav*[Title/Abstract])	4,163,656
#6	#4 OR #5	4,275,104
#7	(program*[Title/Abstract]) OR (intervention[Title/Abstract])	1,870,193
**#8**	**#3 AND #6 AND #7**	**752**

Table S2 Search strategy of Web of Science		
**Number**	**Query**	**Results**
#1	(TS=(esophageal cancer)) OR TS=(oesophageal cancer)	62768
#2	((((((((((TS=(diet*)) OR TS=(nutrition*)) OR TS=(eating)) OR TS=(food)) OR TS=(physical activit*)) OR TS=(exercise*)) OR TS=(walk*)) OR TS=(training)) OR TS=(rehabilitation)) OR TS=(lifestyl*)) OR TS=(behav*)	10062729
#3	(TS=(program*)) OR TS=(intervention)	4029951
**#4**	**#1 AND #2 AND #3**	**1079**

Table S3 Search strategy of Cochrane Library		
**Number**	**Query**	**Results**
#1	MeSH descriptor: [Esophageal Neoplasms] explode all trees	2602
#2	(esophageal cancer):ti,ab,kw OR (oesophageal cancer):ti,ab,kw	5381
#3	#1 OR #2	6033
#4	MeSH descriptor: [Diet] explode all trees	26637
#5	MeSH descriptor: [Exercise] explode all trees	38714
#6	(diet*):ti,ab,kw OR (nutrition*):ti,ab,kw OR (eating):ti,ab,kw OR (food):ti,ab,kw	183759
#7	(physical activit*):ti,ab,kw OR (exercise*):ti,ab,kw OR (walk*):ti,ab,kw OR (training):ti,ab,kw OR (rehabilitation):ti,ab,kw	302674
#8	(lifestyl*):ti,ab,kw OR (behav*):ti,ab,kw	179968
#9	#4 OR #5 OR #6 OR #7 OR #8	545942
#10	(program*):ti,ab,kw OR (intervention):ti,ab,kw	620948
**#11**	**#3 AND #9 AND #10**	**492**

Table S4 Search strategy of Embase (via Elsevier)		
**Number**	**Query**	**Results**
#1	'esophagus cancer'/exp	94242
#2	'esophagus tumor'/exp	114313
#3	'esophageal cancer':ab,ti OR 'oesophageal cancer':ab,ti	41967
#4	#1 OR #2 OR #3	117488
#5	'diet'/exp	445836
#6	'physical activity'/exp	562942
#7	'exercise'/exp	464376
#8	diet*:ab,ti OR nutrition*:ab,ti OR eating:ab,ti OR food:ab,ti OR 'physical activit*':ab,ti OR exercise*:ab,ti OR walk*:ab,ti OR training:ab,ti OR rehabilitation:ab,ti OR lifestyl*:ab,ti OR behav*:ab,ti	5069128
#9	#5 OR #6 OR #7 OR #8	5432735
#10	program*:ab,ti OR intervention:ab,ti	2509444
**#11**	**#4 AND #9 AND #10**	**1240**

Table S5 Search strategy of CINAHL (via EBSCO)		
**Number**	**Query**	**Results**
S1	(MH "Esophageal Neoplasms+")	9,541
S2	(MH "Esophagus+")	6,801
S3	TI esophageal cancer OR TI oesophageal cancer OR AB esophageal cancer OR AB oesophageal cancer	8,611
S4	S1 OR S2 OR S3	18,897
S5	(MH "Diet+")	208
S6	(MH "Nutrition+")	1187
S7	(MH "Exercise+")	1275
S8	TI diet* OR TI nutrition* OR TI eating OR TI food OR TI physical activit* OR TI exercise* OR TI walk* OR TI training OR TI rehabilitation OR TI lifestyl* OR TI behav*	519,844
S9	AB diet* OR AB nutrition* OR AB eating OR AB food OR AB physical activit* OR AB exercise* OR AB walk* OR AB training OR AB rehabilitation OR AB lifestyl* OR AB behav*	914,106
S10	S5 OR S6 OR S7 OR S8 OR S9	1,267,012
S11	TI program* OR TI intervention OR AB program* OR AB intervention	880,523
**S12**	**S4 AND S10 AND S11**	**336**

Table S6 Search strategy of PsycInfo (via EBSCO)		
**Number**	**Query**	**Results**
S1	TI esophageal cancer OR TI oesophageal cancer OR AB esophageal cancer OR AB oesophageal cancer	367
S2	DE "Nutrition" OR DE "Alcoholic Beverages" OR DE "Beverages (Nonalcoholic)" OR DE "Calories" OR DE "Carbohydrates" OR DE "Dietary Supplements" OR DE "Diets" OR DE "Energy Drink" OR DE "Food" OR DE "Mealtimes"	48,311
S3	DE "Physical Activity" OR DE "Actigraphy" OR DE "Exercise"	54,569
S4	DE "Exercise" OR DE "Aerobic Exercise" OR DE"Weightlifting" OR DE "Yoga"	39,403
S5	TI diet* OR TI nutrition* OR TI eating OR TI food OR TI physical activit* OR TI exercise* OR TI walk* OR TI training OR TI rehabilitation OR TI lifestyl* OR TI behav*	408,824
S6	AB diet* OR AB nutrition* OR AB eating OR AB food OR AB physical activit* OR AB exercise* OR AB walk* OR AB training OR AB rehabilitation OR AB lifestyl* OR AB behav*	1,315,713
S7	S2 OR S3 OR S4 OR S5 OR S6	1,370,286
S8	TI program* OR TI intervention OR AB program* OR AB intervention	767,468
**S9**	**S1 AND S7 AND S8**	**29**
